# MAK-4 and -5 supplemented diet inhibits liver carcinogenesis in mice

**DOI:** 10.1186/1472-6882-7-19

**Published:** 2007-06-08

**Authors:** Marialetizia Penza, Claudia Montani, Marija Jeremic, Giovanna Mazzoleni, WL Wendy Hsiao, Maurizio Marra, Hari Sharma, Diego Di Lorenzo

**Affiliations:** 1 Laboratory of Biotechnology, Civic Hospital of Brescia, 25123 Brescia, Italy; 2 Unit of General Pathology and Immunology, School of Medicine, University of Brescia, 25123, Brescia, Italy; 3 School of Chinese Medicine, Hong Kong Baptist University, Hong Kong, China; 4 Department of Gerontological Research, Diabetology Unit-INRCA, Ancona, Italy; 5 Department of Pathology, College of Medicine and Public Health, The Ohio State University, Columbus, 43210-1240 Ohio, USA

## Abstract

**Background:**

Maharishi Amrit Kalash (MAK) is an herbal formulation composed of two herbal mixtures, MAK-4 and MAK-5. These preparations are part of a natural health care system from India, known as Maharishi Ayur-Veda. MAK-4 and MAK-5 are each composed of different herbs and are said to have maximum benefit when used in combination. This investigation evaluated the cancer inhibiting effects of MAK-4 and MAK-5, *in vitro *and *in vivo*.

**Methods:**

*In vitro *assays: Aqueous extracts of MAK-4 and MAK-5 were tested for effects on *ras *induced cell transformation in the Rat 6 cell line assessed by focus formation assay. *In vivo *assays: Urethane-treated mice were put on a standard pellet diet or a diet supplemented with MAK-4, MAK-5 or both. At 36 weeks, livers were examined for tumors, sera for oxygen radical absorbance capacity (ORAC), and liver homogenates for enzyme activities of glutathione peroxidase (GPX), glutathione-S-transferase (GST), and NAD(P)H: quinone reductase (QR). Liver fragments of MAK-fed mice were analyzed for connexin (cx) protein expression.

**Results:**

MAK-5 and a combination of MAK-5 plus MAK-4, inhibited *ras*-induced cell transformation. In MAK-4, MAK-5 and MAK4+5-treated mice we observed a 35%, 27% and 46% reduction in the development of urethane-induced liver nodules respectively. MAK-4 and MAK4+5-treated mice had a significantly higher ORAC value (*P *< 0.05) compared to controls (200.2 ± 33.7 and 191.6 ± 32.2 *vs. *152.2 ± 15.7 ORAC units, respectively). The urethane-treated MAK-4, MAK-5 and MAK4+5-fed mice had significantly higher activities of liver cytosolic enzymes compared to the urethane-treated controls and to untreated mice: GPX(0.23 ± 0.08, 0.21 ± 0.05, 0.25 ± 0.04, 0.20 ± 0.05, 0.21 ± 0.03 U/mg protein, respectively), GST (2.0 ± 0.4, 2.0 ± 0.6, 2.1 ± 0.3, 1.7 ± 0.2, 1.7 ± 0.2 U/mg protein, respectively) and QR (0.13 ± 0.02, 0.12 ± 0.06, 0.15 ± 0.03, 0.1 ± 0.04, 0.11 ± 0.03 U/mg protein, respectively). Livers of MAK-treated mice showed a time-dependent increased expression of cx32.

**Conclusion:**

Our results show that a MAK-supplemented diet inhibits liver carcinogenesis in urethane-treated mice. The prevention of excessive oxidative damage and the up-regulation of connexin expression are two of the possible effects of these products.

## Background

Maharishi Amrit Kalash (MAK) is an herbal formulation composed of two herbal mixtures, MAK-4 and MAK-5. These preparations are part of a natural health care system, known as Maharishi Ayur-Veda [1,2]. MAK-4 and MAK-5 are each composed of different herbs and are said to have maximum benefit when used in combination [3,4]. A large body of research has been published on these two herbal mixtures, including studies which show them to have anticancer, anticarcinogenic, and immune enhancing properties [5-8].

MAK-4 was shown to prevent 7,12-dimethylbenz(a)anthracene (DMBA)-induced mammary carcinoma in rats during both the initiation and promotion phases of carcinogenesis. Also, when the control animals that had developed fully-formed tumors were switched over to a MAK-4 diet, tumor regression was produced in 60% of these animals [9]. In similar experiments, a MAK-5 diet provided protection during the promotion phase of carcinogenesis in rats with DMBA-induced mammary carcinoma, and 60% of control animals with tumor experienced regression of the tumor when switched to the MAK-5 diet [10].

A study on an aggressive lung cancer model, known as Lewis lung carcinoma also showed positive results with the use of MAK-4. Mice with this cancer who were fed with MAK-4 in their diet had a 65% reduction in the number of metastatic nodules and a 45% reduction in the size of the nodules [11]. *In vitro *cell culture experiments on mouse neuroblastoma cells showed that ethanol extracts of MAK-5 produced morphological and biochemical differentiation of 75% of the cells [12], while, in a further recent study, MAK-5 potentiate NGF inducing neuronal differentiation in PC12 cells [13]. In a study reported by the National Cancer Institute (USA), MAK-4 and MAK-5 inhibited processes of neoplastic transformation in rat tracheal epithelial cell lines and human lung tumor cell lines [14]. At molecular level, MAK-4 increases levels of mRNA transcripts of two genes that code for hepatic glycosyltransferases in the rat. Distinct changes were observed in glycosyltransferases, in the rat hepatic nodules from which cancer arises [15].

The scope of the present investigation is to evaluate the cancer inhibiting effects of MAK-4 and MAK-5 *in vitro *and *in vivo*, and to investigate the possible mechanism of their action. Extracts of MAK-4 and MAK-5 were tested for their effects on *ras*-induced cell transformation in the Rat 6 cell line, which has been extensively used to study the effect of various chemicals on the *ras *oncogene expression [16]. Molecular changes that underpin tumor formation have been shown to involve activation of *ras *in different tissues of humans and animals [17-20].

The *in vivo *portion of the study was conducted on liver cancer-susceptible strains of mice [19]. The effect of MAK-4 and MAK-5 on the prevention of hepatoma induced by urethane in CH3HeJ, F1 hybrid mice was evaluated.

Free radicals have been implicated in carcinogenesis [21-24]. Antioxidants, which provide protection against free radical damage, are present in MAK 4 and MAK-5. The chemical composition of these herbal mixtures includes alpha tocopherol, beta-carotene, ascorbate, bioflavonoid, catechin, polyphenols, *trans *resveratrol, riboflavin, tannic acid, and other low molecular weight substances [25]. Therefore, we have evaluated several parameters of antioxidant defense in the animals. We have performed assays to measure the oxygen radical absorbance capacity (ORAC) and activities of the enzymes glutathione peroxidase (GPX), glutathione-S-transferase (GST), and NAD(P)H: quinone reductase (QR).

Connexins are integral membrane proteins constitutive of gap junctions (gj), ubiquitous intercellular channels that allow the direct and selective cell-to-cell exchange of small cytoplasmic molecules and ions. These exchanges were long thought to play an important role in the regulation of cell growth, differentiation and functions in a number of tissues [26]. Connexins comprise a multigenic family, with each member presenting its own tissue-specific distribution and whose expression is developmentally regulated [27]. For quite a long time the loss or the alteration of gap junction intercellular communication (GJIC) was proposed to play an important role in the process of carcinogenesis [28], in particular, when occurring in animal models as well as in human during hepatocarcinogenesis [29,30] and after animal exposure to tumor promoting agents [31].

Several *in vitro *studies have shown a correlation between the anticarcinogenic potential of antioxidant molecules and their ability to up-regulate GJIC and connexin (cx) expression [32], being these effects cell-type specific as well [33]. Therefore, the effect of MAK on the expression of liver-specific members of the connexin family of proteins, namely cx32, cx26 and cx43 [34] was also analysed.

## Methods

### Chemicals

Trolox (6-hydroxy-2,5,7,8-tetramethylchroman-2-carboxylic acid) and 1-chloro-2-4-dinitrobenzene were obtained from Aldrich Chemical Co. (Milan, Italy). The Chelex 100 resin (200–400 mesh, Na form) was purchased from Mallinckrodt Inc. (St. Louis, Mo, USA). The Ransel kit was obtained from Randox (Crumlin, UK). All the other reagents were obtained from Sigma Chemical Co. (Milan, Italy) unless otherwise indicated.

### Herbal mixtures

Maharishi Amrit Kalash-4 (MAK-4) and -5 (MAK-5) were gifts from the Global Trading Group (Verona, Italy). MAK-4 is in paste form and contains the following ingredients: Raw sugar, Ghee, *Terminalia Chebula*, *Emblica Officinalis*, *Hooney*, *Cinnamon*, *Cyperus Rotundus*, *Curcuma*, *Piper Longum*, *Santalum Album*, *Bacopa Monnieri*, *Cyperus Scariosus*, *Mesua Ferrea*, *Convolvulus Pluricaulis*, *Licorice*, *Embelia Ribes*. MAK-5 is in tablet form and contains the following ingredients: *Sphaeranthus indicus*, *Gymnema aurentiacum*, Withania Somnifera, *Licorice*, *Ipomea Digitata*, *Asparagus Ascendens*, *Acacia Arabica*, *Emblica Officinalis*, *Tinospora Cordifolia*, *Asparagus Racemoso*, *Vitex Trifolia*, *Convolvulus Pluricaulis*, *Curculigo Orchioides*, *Capparis Aphylla*, *Argyrea Speciosa*, *Vanda spatulatum*, *elephant creeper*.

Qualitative chemical analysis has revealed that MAK-4 and MAK-5 contain multiple antioxidants, including alpha-tocopherol, beta-carotene, ascorbate, bioflavonoid, catechin, polyphenols, riboflavin, tannic acid, and other low molecular weight substances [25]. The quantitative analysis of these constituents has not been done. The total polyphenols were assessed by the reduction of phosphotungsticphosphomolybdic acids (Folin Ciocalteu's reagent) by phenols in alkaline solution [35] and sodium carbonate method. Their concentration in crude dried material is 21,76 mg per gram. MAK-4 contains 7 μg per dried gram of crude material of trans-resveratrol determined by high performance liquid chromatography. The total antioxidant activity of MAK-4, measured by a modification of the Ferric Reducing Ability of Plasma (F.R.A.P) assay is 144 μmol per gram of dried crude material. The F.R.A.P assay measures the percentage of FeIII (FeCl3.6H20) reduced to FeII by water extractable antioxidants. Reagents were prepared as indicated by Iris et al. [36]. The production of MAK-4 and MAK-5 is subjected to stringent quality control measures, to assure uniform composition and quality from batch to batch.

### Preparation of MAK extracts

Aqueous extracts of MAK-4 or MAK-5 were prepared by adding 2 mg of the herbal mixture to one ml of near-boiling double distilled water and stirring for 20 minutes. The suspension was spun at 6000 rpm for 20 minutes, then the supernatant was collected and filtered with a sterile filter. The appropriate amount of this stock solution was then mixed with 2× Dulbecco's Modified Eagle Medium (DMEM) and double distilled water to prepare a 1× DMEM medium with the designated dosages of MAK extract for later usage.

### Cell cultures

The Rat 6 cell line is a subclone of the Fisher rat embryo fibroblast, originally derived by Freeman and colleagues [37]. All stock cell lines were grown in 90 mm dishes in DMEM supplemented with 10% calf serum (D10CS) (Gibco/BRL Co. USA). Cultures were maintained in a humidified incubator at 37°C with 5% CO_2 _in air, and fed twice a week with fresh DMEM plus D10CS.

### Cytotoxicity

Rat 6 cells and T24-transformed Rat 6 cells (R6/T24) were seeded in triplicate at 10^4 ^cells per 60 mm plate in D10CS with the aqueous extracts of MAK-4 and MAK-5. MAK-4 and MAK-5 were first tested at the dosage ranging from 200 to 1000 μg/ml as designated. The cells were maintained in the tested mixtures for 5 days. At the end of the treatment, the cells were trypsinized and counted by Coulter Counter. Cytotoxicity was expressed as percent of cell survival compared to the untreated control.

### Transfection and focus formation assay

The DNA-mediated transfection procedure was essentially based on the method of Bacchetti and Graham [38] and Wigler et al, [39] with slight modifications by Hsiao et al. [16]. Plasmid pT24 containing a 6.4-kilobase BamHI fragment corresponding to the cellular coding sequence of the mutated human bladder cancer c-H-*ras *oncogene was obtained from Goldfarb et al. [40] and used for the transfection assay. The recipient cells were seeded at 5 × 10^5 ^per 90 mm plate in D10CS. One day later, the cells were fed with fresh D10CS, 4 hours before transfection. Calcium phosphate-precipitated DNA (1 μg of plasmid DNA plus 20 μg of carrier DNA) was added to each dish and incubated for 4 hours at 37°C. The medium was then removed and the transfected cells were fed with fresh D10CS. The next day, the cultures were trypsinized and re-plated in DMEM plus D5FCS (Foetal Calf Serum) at approximately 2.5 × 10^5 ^cells per 90 mm plate. The replated cultures were fed twice a week with D5FCS throughout the remainder of the experiment. To determine the effects of MAK-4 and MAK-5 on *ras *induced foci formation, aqueous extracts of the herbal mixtures were added to the transfected cultures one day after replating and throughout the remainder of the experiment. Three to 4 weeks after transfection, the plates were stained with Giemsa stain and scored for transformed foci.

### Animals and treatments

Female C3H/HeN mice and male C57BL/6J mice were purchased from Charles River (Milan, Italy). C57BL/6J males were crossed with C3H/HeN females to obtain CH3-HeJ hybrid mice (a strain of mice with high susceptibility to liver and lung tumorigenesis induced by various means [41,42]. Only male hybrid mice were used for the experiments. In one experiment, a group of 85 hybrid mice was treated at 8 days of age with a single dose of urethane diluted in 0.9% sodium chloride solution, at the dosage of 300 mg/kg body weight. After weaning, the mice were separated and put on different diets; 25 mice were fed a standard pellet diet and 20 mice/group were fed a pellet diet containing: MAK-4 (4%, w/w) MAK-5 (1%, w/w) and a combination of MAK-4+5 at the same concentrations (diets were prepared by adding commercial preparations of MAK-4 and MAK-5 to the pellet mixture, Piccioni, Milan). The dose of MAK-4 and MAK-5 was extrapolated from the recommended human dose, which is 10 g MAK-4 twice a day and 500 mg MAK-5 twice a day. All mice had tap water *ad libitum *and were kept under observation in the same room. All the surviving animals were sacrificed at 36 weeks of age and their livers collected for histological analysis.

A separate experiment was conducted on five groups of mice (10 mice/group). Two groups of mice were handled in the same manner as described above (+/- urethane) and three groups of mice fed a diet supplemented with the three combinations of MAK. At 36 weeks of age the animals were sacrificed and their livers and sera collected for biochemical analysis. The serum samples were stored at -80°C and the livers were stored in liquid nitrogen until the time of analysis.

A time-course experiment was conducted on groups of 3-month-old hybrid mice. The mice were fed the diet supplemented with 4% (w/w) MAK-4 and 1% (w/w) MAK-5 or both, for 3 days, 7 days, 2 weeks, 1 month, or 3 months. The mice were sacrificed at their respective times and their livers and sera collected for biochemical analysis. The serum samples were stored at -80°C and the livers were stored in liquid nitrogen until the time of analysis.

A separate time-course experiment was conducted on CH3-HeJ healthy 3-month-old hybrid mice. The mice were fed with the MAK-supplemented diet for 1 day, 1 week, 1 month, 2 months, or 3 months. A control group of CH3-HeJ mice was fed with a standard pellet diet. The mice were sacrificed at their respective times and their livers were collected for immunoblot analysis.

The procedures involving the animals and their care were conducted in accord with institutional guidelines, which comply with national and international laws and policies [National Institutes of Health, Guide for the Care and Use of Laboratory Animals, 1996 (7th edition) (Washington, D.C.); National Academy Press, National Research Council Guide] [43]. Mice were kept in animal rooms maintained at a temperature of 23°C, with natural light/dark cycles.

### Liver tumors

The mice were sacrificed at 36 weeks of age. Their livers were examined for gross lesions, fixed in formaline, embedded in paraffin, cut, and stained with hematoxylin and eosin for histopathological examination. For each mouse, all the visible nodules were counted in two sections, one from the left liver lobe and one from the median liver lobe, and the size of the nodules was measured in cubic millimetres (mm^3^).

### Oxygen radical absorbance capacity of serum

After sacrifice at 36 weeks, the hybrid mice sera were analyzed for oxygen radical absorbance capacity (ORAC) using a modified method of Cao et al. [44]. The reaction mixture for the ORAC assay contained 1.67 × 10^-8 ^M beta-Phycoerythrin (β-PE), 0.3% H_2_O_2_, and 9 × 10^-6 ^M CuSO_4 _in 7.5 × 10^-2 ^M phosphate buffer, pH 7.0 (200 ml). The phosphate buffer was passed through Chelex 100 resin before it was used to prepare the solutions, and was used as a blank in the assay. The reaction mixture and sera were pipetted into fluorimetry 96-well microplates. Trolox, a water-soluble vitamin E analogue, was used as a control standard. Samples containing β-PE alone (1.67 × 10^-8 ^M in phosphate buffer) were also prepared to monitor the spontaneous decay of fluorescence of this indicator protein under the experimental conditions. H_2_O_2 _and CuSO_4 _were added to each well to generate hydroxyl radicals (OH°), then the microplate was put into the pre-warmed plate (37°C) of a Titertek Fluoroscan II (Flow Laboratories, Milan, Italy) and the fluorescence of β-PE was measured every five minutes until zero fluorescence occurred, using the excitation and emission wavelengths of 540 nm and 565 nm, respectively. All fluorescence readings were automatically corrected for the spontaneous decay of β-PE.

The ORAC values of the mice sera were calculated by measuring the net protection area (S) under the quenching curve of β-PE in the presence of the sera. One ORAC unit was designated as the net protection provided by 1 μM (final concentration) Trolox. The ORAC units of a sample were calculated as follows: ORAC units = (Ssample-Sblank)/(S1μM Trolox-Sblank).

### Liver cytosolic enzyme activities

Liver homogenates were analyzed for activities of the following enzymes: glutathione peroxidase (GPX), glutathione-S-transferase (GST), and NAD(P)H: quinone reductase (QR). Homogenates were prepared in a medium consisting of 140 mM KCl and 25 mM potassium phosphate buffer (pH 7.4), and centrifuged at 6000 × g for 10 minutes. The protein concentration of the samples (crude extracts) was determined by the method of Bradford [45].

GPX activity was determined using the commercial Ransel kit which is based on the principle of NADPH oxidation; GPX catalyzes the oxidation of glutathione by cumene hydroperoxide. In the presence of glutathione reductase and NADPH, the oxidized glutathione is converted to the reduced form with a concomitant oxidation of NADPH to NADP+. The decrease in absorbance was measured spectrophotometrically at 340 nm. For each determination, samples containing 5 μg liver protein were used.

GST activity was assayed as previously reported [46]. Briefly, a solution of 1 mM glutathione and 1 mM 1-chloro-2-4-dinitrobenzene in 0.1 M phosphate buffer, pH 6.5, was added to samples containing 10 μg of liver protein. The increase in absorbance was read spectrophotometrically after 1, 2, and 3 minutes at 340 nm.

QR activity was measured as described previously [47]. Briefly, 420 μl of a mixture containing 25 mM Tris, pH 7.4, 0.7 mg/ml bovine albumin, 0.01% Tween 20, 1.07 mM glucose-6-phosphate, 2.15 U/ml glucose-6-phosphate dehydrogenase, 5.35 μM FAD, 32 μM NADP, and 321 mg/l MTT were added to 50 μl of sample. After the addition of 5 mM menadione in acetonitrile (10 μl/ml), the reaction was carried on for 5 minutes and the absorbance read spectrophotometrically at 610 nm. In order to determine the proportion of MTT reduction attributable to QR, another set of samples was pre-treated in the presence of 0.3 mM dicoumarol in 0.5% DMSO and 5 mM potassium phosphate (pH 7.4) and assayed as described above. For each determination, samples containing 150 μg liver protein were used.

### Measurement of connexin expression by immunoblot analysis

The influence of the MAK-supplemented diet on the expression of the three liver-specific connexins (cx26, cx32, and cx43), was measured. For the immunoblot analysis, the Western blot technique was used. Fragments of liver from control mice fed a normal diet or mice fed the MAK-supplemented diet for 1 day, 1 week, 1 month, 2 months, or 3 months were prepared and alkali-extracted [30]. Alkali-resistant membrane proteins, corresponding to approximately 15 μg of total protein/sample, were resolved on 12.5% SDS-PAGE [48] and then transferred onto a nitrocellulose membrane (Schleicher and Schuell, Keene, NH, USA). As a control for normalizing the amount of samples, the gels were stained with 0.25% Comassie Blue solution (R 250/G 250, 1:1) (Bio Rad Laboratories, Hercules, CA, USA).

Membranes were hybridized with mouse anti-cx26 (1:750) (Zymed Laboratories Inc., San Francisco, CA, USA), anti-cx32 (1:1000) (Chemicon International Inc. Temecula, CA, USA), or anti-cx43 (1:1000) (Chemicon International Inc. Temecula, CA, USA) monoclonal antibodies, followed by reaction with peroxidase-conjugated anti-mouse IgG (1:2000) (Amersham International, Buckinghamshire, UK). Immunopositive reaction was detected by the enhanced chemiluminescence (ECL, Amersham, Buckinghamshire, UK) method.

### Statistical analysis

Statistical analysis was performed by Two Way ANOVA Test followed by Post Hoc Bonferroni analysis.

## Results

### Effect of MAK-4 and MAK-5 on H-ras-induced cell transformation assessed by focus formation assay

MAK-4 and MAK-5 were initially tested for their inhibitory effects in H-*ras*-induced cell transformation at 500 μg/ml in Rat 6 cells (Table 1). MAK-4 exerted no or slightly enhancing effect on the formation of transformed foci, while MAK-5 exhibited 92% reduction compared to the untreated control.

**Table 1:** Effect of MAK-4 and MAK-5 on *H-ras*-induced cell transformation in Rat 6 cells

**Treatment**	**Exogenous H-*ras *gene**	**Cytotoxicity (% survival)**	**Foci/plate ± SD**	Relative number of foci**
None	-	100	0	0.0
None	+	100	15.3 ± 2.5	1.00
MAK-4 (500 μg/ml)	+	79	13.0 ± 1.7	0.84
MAK-5 (500 μg/ml)	+	73	1.2 ± 1.3	0.078

Rat 6 cells transfected with an activated H-*ras *oncogene were replated in D5FCS growth medium at ~2.5 × 10^5^/90 mm plates as described in Materials and Methods. The treatment groups were fed with D5FCS plus the herbal extracts one day after replating and throughout the remainder of the experiment. All cultures were fed twice a week. At day 18 from the time of transfection, the cultures were fixed with 10% formaldehyde, stained with Giemsa stain, and scored for transformed foci by focus formation assay. Triplicate plates were used for each experimental group. Cytotoxicity was based on cell numbers of each treatment group counted by Coulter Counter and expressed as percent of cell survival compared to the untreated control. ** The relative number of foci is the number of foci obtained from the treatment groups divided by those obtained from the no-treatment group.

MAK-4 and MAK-5 were slightly toxic. At 500 μg/ml, the survival rate of the MAK-4 and MAK-5 treated cells were 83% and 69% respectively compared to controls.

MAK-4 and MAK-5 were also tested at 200, 500, and 1000 μg/ml (Table 2). MAK-5 was inhibitory on the formation of H-*ras*-foci, while MAK-4 seemed to enhance cell transformation. The enhancement effect was most prominent when cultures were treated with a low dosage of MAK-4.

**Table 2:** Dosage effect of MAK-4 and MAK-5 on *H-ras*-induced cell transformation in Rat 6 cells

**Treatment**	**Exogenous H-*ras *gene**	**Cytotoxicity (% survival)**	**Relative number of foci**
Mock control	-	100	0
None	+	100	1.0

MAK-4:			
200 μg/ml	+	132	1.75
500 μg/ml	+	98	1.29
1000 μg/ml	+	92	1.35

MAK-5:			
200 μg/ml	+	135	0.69
500 μg/ml	+	64	0.28
1000 μg/ml	+	48	0.26

Data were obtained from two individual experiments by focus formation assay. Triplicate plates were used for each experiment. Experimental procedures were the same as indicated in Table 1.

To test the combinatory effect of MAK-4 and MAK-5, 500 μg/ml of MAK-5 were tested alone or in the presence of 200 and 500 μg/ml of MAK-4. As reported in Table 3, although MAK-4 alone showed no effect on H-*ras*-foci, it slightly enhanced the inhibitory effect of MAK-5 by 10%.

**Table 3:** Combinatory effect of MAK-4 and MAK-5 on H-*ras*-induced transformation assessed by focus formation assay

**Treatment**	**Exogenous H-*ras *gene**	**Cytoxicity (% survival)**	**Foci/plate ± SD**	**Relative number of foci**
Mock control	-	100	0	-
None	+	100	81.7 ± 2.3	1.00
MAK-5, 500 μg/ml *plus *MAK-4, 0 μg/ml	+	65	25.3 ± 2.7	0.52
MAK-5, 500 μg/ml *plus *MAK-4, 100 μg/ml	+	81	17.7 ± 0.9	0.28
MAK-5, 500 μg/ml *plus *MAK-4, 200 μg/ml	+	88	20.3 ± 0.8	0.31

The experimental procedure and the duration of treatment was the same as Table 1.

### Effect of MAK-4 and MAK-5 on the growth of normal and transformed Rat 6 cell lines

To determine whether or not the inhibition on *ras*-foci was due to a differential killing of *ras*-transformed cells, MAK-4 and MAK-5 were tested for their cytotoxic effect on a fully established clonal *ras *transformed cell line R6/T24. In the same experiment, normal Rat 6 cells were also included in the test (Table 4). MAK-4 and MAK-5 slightly stimulated the growth of Rat 6 at 200 μg/ml, while exerted mild toxicity at 500 μg/ml. At 1000 μg/ml, the cytotoxic effect of MAK-5 became evident. Rat 6 cells were more sensitive than the transformed R6/T24 to MAK-5 cytotoxicity (Table 4). This result suggests that the inhibitory effect of MAK-5 extract on foci formation is not a result of direct cell killing of the transformed cells, instead, it may exert its effect at an early stage of *ras*-induced transformed foci.

**Table 4:** Dosage effect of MAK-4 and MAK-5 on the growth of transformed Rat 6 cells

**Treatment**	**Cells (× 10^6^/60 mm plate) ± SD**		**% of survival**	
	**Rat 6**	R6/T24*	**Rat 6**	**R6/T24**
**None **(control)	0.94 ± 0.04	1.32 ± 0.04	100	100

**MAK-4**:				
200 μg/ml	1.24 ± 0.12	1.52 ± 0.05	138	141
500 μg/ml	0.94 ± 0.08	1.27 ± 0.06	112	115
1000 μg/ml	0.82 ± 0.09	1.29 ± 0.07	91	119

**MAK-5**:				
200 μg/ml	1.57 ± 0.09	1.77 ± 0.09	119	164
500 μg/ml	0.84 ± 0.08	1.07 ± 0.04	84	101
1000 μg/ml	0.58 ± 0.06	0.73 ± 0.01	66	79

R6/T24 is a permanent cell line transformed by an activated human c-H-*ras *oncogene. Both normal and H-*ras *transformed Rat 6 cells were seeded in triplicate at 10^4 ^cells per 60 mm plate in D10CS. Next day, cultured were switched to D5FCS, or FCS plus herbal extracts at designated dosages for 5 days. At the end of the treatment, cells were trypsinized and counted by Coulter Counter. Cytotoxicity was expressed as percent of cell survival compared to the untreated control.

### Liver tumors

**Figure 1:**
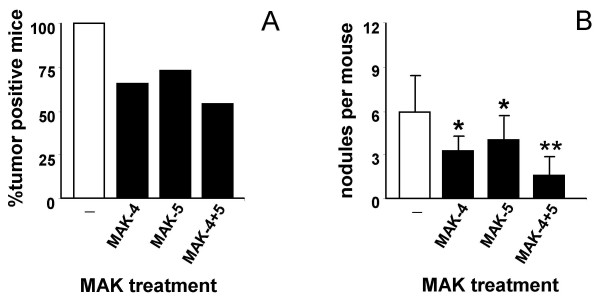
**Histological analysis of mice livers**. Hybrid F1 mice were injected with urethane (300 mg/kg) at 8 days of age. A group was then put on a MAK-supplemented diet, another group was fed a standard pellet diet. At 36 weeks of age the mice were sacrificed and the livers examined for the presence of tumors mouse (Panel A) and for the number of nodules per mouse (Panel B) (* p < 0.05, ** P < 0.001). Statistical analysis was performed by Two Way ANOVA Test followed by Post Hoc Bonferroni analysis.

The effect of MAK-4 and MAK-5 was tested *in vivo *in CH3-HeJ hybrid male mice as indicated in materials and methods. There was a significant reduction in the number of mice that developed liver tumor nodules in the MAK-fed groups, compared to controls. All the 34 mice that were treated with urethane and fed the normal diet developed liver tumors at the age of 36 weeks, whereas only 54% of the urethane-treated mice (69 mice) on the diet supplemented with MAK showed nodule formation (23 mice on MAK-4, 22 on MAK-5 and 24 on MAK-4+5) (Figure 1, **Panel A**).

Among the tumor-positive mice, the number of nodules was significantly lower in the groups fed with combination of MAK-4+5 (3.2 ± 1.9, 4.0 ± 2.2, 1.8 ± 1.0 *vs. *6.1 ± 3.0 nodules/mouse, respectively) (Figure 1, **Panel B**). The average size of the nodules was similar in the MAK-fed and control groups (data not shown).

### Oxygen radical absorbance capacity (ORAC)

**Figure 2:**
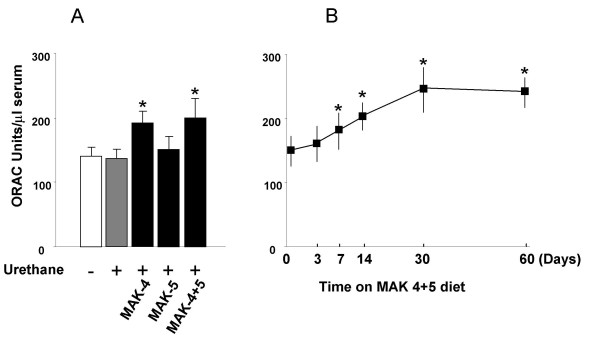
**Oxygen radical absorbance capacity (ORAC)**. Oxygen radical absorbance capacity (ORAC) in urethane-treated mice on MAK-supplemented diets, urethane-treated mice on a normal diet, and control mice on the normal diet (Panel A). The time-course experiment was performed on healthy male mice put on the MAK4+5-supplemented diet (for 0, 3 days, 1 week, 2 weeks,1 month, or 2 months) without any previous treatment (Panel B). One ORAC unit represents the equivalent ORAC activity of 1 μmol Trolox. (* P < 0.05). Each group of animals was composed of 10 mice. At 36 weeks of age the mice were sacrificed, the serum collected and stored at -80°C until the day of the assay. Data are reported as mean ± SEM of three experiments. Statistical analysis was performed by Two Way ANOVA Test followed by Post Hoc Bonferroni analysis.

The ORAC value was significantly higher (*P *< 0.05) in the urethane-treated mice fed a diet supplemented with MAK-4 and a combination of MAK-4+5, compared to the controls (Figure 2, **Panel A**).

Time-course experiments performed in the 3-month-old hybrid mice fed with the effective MAK-4+5 combination, showed a progressive increase in ORAC which reached significance at 7 days after starting the MAK-supplemented diet. The ORAC value reached its maximum at 1 month and this level was maintained for the course of the experiment (Figure 2, **Panel B**).

### Liver cytosolic enzyme activities

**Figure 3:**
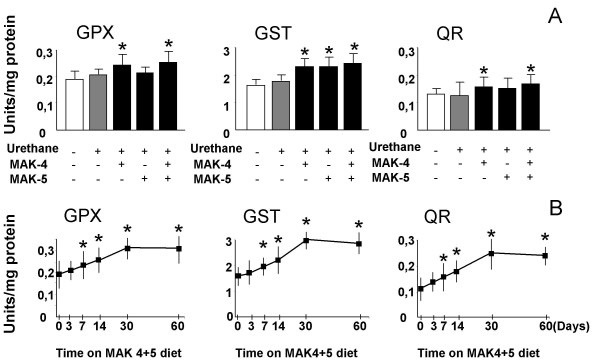
**Enzyme activities in mice**. Enzyme activities in mice treated with urethane plus MAK-supplemented diets, urethane-treated mice on a normal diet, and control mice on the normal diet. (Panel A). The time-course experiment was performed on healthy male mice put on the MAK-4+5 supplemented diet (for 3 day, 1 week, 2 weeks,1 month, or 2 months) without any previous treatment. Each group was composed of 10 mice. At 36 weeks of age the mice were sacrificed, the livers collected and stored in liquid nitrogen until the day of the assay. Data are reported as mean ± SEM of several experiments. (* *P *< 0.05 and ** *P *< 0.001). Statistical analysis was performed by Two Way ANOVA Test followed by Post Hoc Bonferroni analysis.

Liver homogenates were analysed for activity of the enzymes: Glutathione peroxidase (GPX) activity was higher in homogenates of the urethane-treated mice fed a diet supplemented with MAK-4, MAK-5 and MAK-4+5, compared to controls. Glutathione-S-transferase (GST) was significantly higher in all the groups treated with MAK and NAD(P)H: quinone reductase (QR) activity was higher in the mice fed with MAK-4 and MAK4+5, compared to controls (Figure 3, **Panel A**).

Results of a time-course experiments in 2-month-old hybrid mice fed with the most effective combination MAK-4+5 showed a progressive increase in the activities of GPX, GST, and QR, which began to be detectable after 1 day on the MAK-supplemented diet. A significant increase (*P *< 0.05) was seen at 1 week for all the samples. The highest levels were reached at 1 month for all three enzymes (Figure 3, **Panel B**).

### Expression of gap junction proteins in the liver

**Figure 4:**
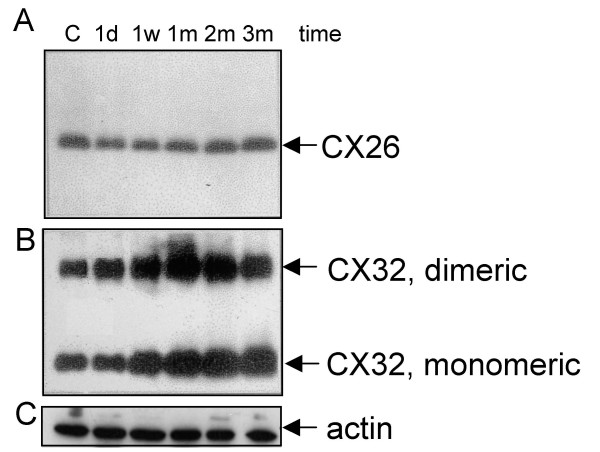
**Western blot analysis of gap junction proteins cx26 and cx32 in mouse liver**. Equal amounts of alkali-resistant membrane proteins (corresponding to 15 μg total lysate) were loaded in each lane. The same membrane was probed with anti-cx26 and anti-cx32, subsequently. Lanes: normal diet-fed (control: C); 1 d (1 day); 1 w (1 week); 1 m (1 month); 2 m (2 months);3 m (3 months) of MAK-4+5 supplemented diet. Panel A: Anti-cx26 antibody; a 21 kDa band indicates the presence of cx26 protein. Panel B: Anti-cx32 antibody; two bands of 27 kDa and 48 kDa were detected corresponding, respectively, to the monomeric and dimeric forms of the cx32 protein. Panel C: Anti-actin antibodies hybridization. Results are representative of three independent immunoblots.

We than measured the influence of the MAK-4+5 combination on the expression of the three liver-specific connexins (cx26, cx32, and cx43). The level of cx26 expression was similar in all the groups of mice treated with the MAK-supplemented diet and in the control (Figure 4, **Panel A**). A significant, time-dependent increase in cx32 was observed in the liver of all the groups of MAK treated mice compared to the normal diet-fed controls. Cx32 expression increased 2-fold after 1 week of treatment, and 3- to 4-fold at 3 months (Figure 4, **Panel B**). The anti cx32 antibody reacted with two antigens with molecular masses of 27 kDa and 48 kDa. The 48 kD band was identified as a stable dimeric form of cx32, as previously reported [49].

## Discussion

The present study reveals the ability of MAK-4 and MAK-5 to inhibit cell transformation induced by *ras *oncogene in an *in vitro *cell system and *in vivo *liver carcinogenesis in mice.

The group of mice fed a diet supplemented with a combination of MAK-4 and MAK-5, had a 46% reduction in the number of liver nodules, compared to mice that were fed a normal diet. Among the tumor-positive mice, the number of tumor nodules was significantly lower in the MAK fed mice. The ability of MAK-4 and MAK-5 to inhibit carcinogenesis may be due to several different mechanisms that may be difficult to identify. The complex mixture of these preparations will not allow the identification of effects due to single components, until extract fractionation is performed. Here we have investigated the ability of MAK-4 and MAK-5 to scavenge free radicals and reactive oxygen species (ROS) and regulate connexins, which are markers of tissue integrity and tumor formation. Free radical-mediated reactions such as lipid peroxidation are implicated in carcinogenesis [21] and there is increasing evidence that the initiation and promotion phases of cancer is favoured by free radical reactions [22-24]. Thus, antioxidants which act to control the oxidative state may represent a major line of defense against cancer development. Multiple antioxidants are present in MAK-4 and MAK-5 such as alpha-tocopherol, beta-carotene, ascorbate, bioflavonoid, catechin, polyphenols, riboflavin, and tannic acid and many others that are still uncharacterized [25]. A Previous research has shown that these mixtures of chemicals scavenge free radicals and ROS such as superoxide, hydroxyl, and peroxyl radicals, and hydrogen peroxide generated in both cellular (neutrophil) and noncellular (xanthine-xanthine oxidase) systems [50], in a dose-dependent manner. MAK-4 and MAK-5 inhibit the *in vitro *peroxidation of lipids in rat liver microsomes [51,52], reduce lipid peroxide levels and inhibit the oxidation of low-density lipoprotein (LDL) [53-55].

In the present work, the antioxidant properties of MAK-4 and MAK-5 were confirmed *in vivo *in mice. The oxygen radical absorbance capacity (ORAC) assay measured a significantly higher value in mice fed the MAK supplemented diet, which increased progressively from day 7 and reached a maximum level at 1 month that was maintained throughout the course of the experiment.

Another line of antioxidant defense was evaluated by measuring the activity of the liver enzymes GPX, GST and QR. All these enzymes were upregulated in the MAK-fed mice. It is known that many compounds that block toxic, mutagenic, and neoplastic effects of carcinogens have in common the ability to elevate levels of phase II detoxification enzymes such as GST and QR [56]. Inducers of phase II enzymes, present in fruits and vegetables, have been shown to prevent carcinogenesis [57]. Substantial evidence has accumulated to suggest that the induction of phase II enzymes is a causal mechanism for protection since these enzymes divert ultimate carcinogens from reacting with critical cellular macromolecules [57]. Epidemiological evidence links a diet rich in antioxidants from fruits and vegetables with a low risk of cancer [58]. However, the optimal intake of antioxidants may not be achievable by diet alone: natural compounds and natural food supplements may be of help to reach an effective antioxidant protection, particularly in subgroups of the population at high risk of developing cancer. Accumulating evidences suggest that several antioxidant compounds, irrespective of their structure, may counteract the promotive action of chemicals by interfering with the oxidant-dependent down-regulation of gap junction function [59]. This possibility was explored by evaluating the effect of MAK on liver specific connexins expression. Gap junctions are specialized intercellular membrane in which connexins are regulated developmentally and in a tissue-specific manner [27]. Altered connexins expression has been proved to occur during the carcinogenic process [28]. Since restoration of normal GJIC appears to work as tumor suppressive element, GJIC is being exploited as end point for identification of cancer chemopreventive agents [28]. *In vivo*, hepatocytes express at least three different connexins, cx32, cx26 and cx43 [34]. Cx32 levels were much higher in the MAK-fed mice compared to the control. These findings indicate that MAK enhance gap junctional communication in liver of CH3-HeJ mice and may support previous *in vitro *findings which demonstrate that oxygen radical scavengers induce liver-specific cx expression in cultured hepatocytes [59]. Thus the antioxidant induced up-regulation of cx expression may be considered as a possible mechanism for the cancer-preventive properties of MAK.

The *in vitro *studies here reported show that MAK-5 produced a 92% reduction in the *ras*-induced cell transformation. MAK-4 did not have the same effect: on the contrary it showed a slight enhancement of cell transformation. Both MAK-4 and MAK-5 were found to be slightly toxic to the Rat 6 cell line; however, they had no apparent toxic effect on the *ras*-transformed cell line R6/T24. It is therefore very unlikely that the inhibitory effect of the MAK-5 extract on foci formation is the result of direct cell killing of the transformed cells, but instead it may exert its effect on the process of transformation by the H-*ras *oncogene. The discrepancy between the *in vitro *and *in vivo *data, with regard to their anticarcnogenic properties, might be due to the fact that the *in vitro *experiments were performed with a water soluble fraction of MAK-4 and -5. Extraction may have out selected fractions of several chemicals that are important for the activity of MAKs, which relative solubility in not known.

Literature data indicate that another possible mechanism for the anticarcinogenic properties of MAK-4 and MAK-5 is the enhancement of immunity, that has been demonstrated in several previous studies in which animals treated with MAK-5 had a 32–88% increase in mitogen-induced lymphocyte proliferation. This enhanced immune functioning lasted as long as 15 days after discontinuation of MAK-5 treatment. Macrophage superoxide anion generation and phagocytosis were unchanged during MAK-5 treatment, indicating that the protective abilities of the macrophages were unaltered [60]. In a separate study, lymphocytes from mice that were fed MAK-5 in their diet had higher proliferative responses to activation by phytohemagglutinin and anti-CD3 antibodies. The spontaneous rate of lymphocyte proliferation was not increased by MAK-5. Peritoneal macrophages from the mice fed MAK-5 showed an increase in tumor cell killing, when activated with either interferon or lipopolysaccharide. Moreover, activated macrophages from MAK-5-fed mice produced significantly more nitric oxide than control animals. Nitric oxide is considered an important mediator in the tumoricidal and bactericidal actions of macrophages. Both nitric oxide production and cytotoxicity were inaltered by MAK-5 in the inactivated macrophages [6]. Hence, MAK-5 induces *in vivo *priming of both T cells and macrophages, resulting in enhanced functioning of the immune system.

Immunological studies conducted by other groups confirmed these observations [8]. These authors showed that MAK-4 has similar immune-enhancing properties as MAK-5 [8]. In addition, MAK-4 significantly increased stimulated splenic production of interleukin-2, that is involved in the regulation of the immune system. Another group investigated the dose-dependent activation of immune functioning in mice treated with MAK-5. They showed that the daily assumption of MAK-5 at the dose of 50 mg/kg, enhanced macrophage functioning as well as lymphocyte responsiveness [61]. Altogether these studies indicate that the anticarcinogenic activities of MAK may also be a result of enhanced functioning of the immune system.

## Conclusion

The present study demonstrates that MAK-4 and MAK-5 inhibit liver carcinogenesis in mice. These herbal mixtures also increase antioxidant defenses and liver-specific connexin expression, both of which may be involved in the mechanism of action of the anticancer potential of these compounds.

## Competing interests

The author(s) declare that they have no competing interests.

## Authors' contributions

MP and CM: animal treatments, liver cytosolic enzyme activities and evaluation of liver tumor formation. MJ: Animal breeding, colonies expansion, treatments and sacrifice. Tumor tissue analysis. GM: expression of gap junction proteins. MM: oxygen radical adsorbent capacity assay. DDL and HS: results evaluation. WH: H-ras induced cell transformation, focus formation assay and evaluation of the growth rate and transformed cells. All authors read and approved the final manuscript.

## Pre-publication history

The pre-publication history for this paper can be accessed here:


